# Standalone Microthane® breast implants in the prepectoral position: Positive outcomes from combining technical and surgical advantages supported by patient-reported outcomes

**DOI:** 10.1016/j.jpra.2025.10.021

**Published:** 2025-10-21

**Authors:** Edoardo Bruno, Matteo Cilluffo, Andrea Columpsi, Camilla Cavaliere, Francesco Cavaliere, Lucio Fortunato, Diego Ribuffo, Andrea Loreti

**Affiliations:** a Department of Surgery ''P. Valdoni'' - Unit of Plastic and Reconstructive Surgery, Policlinico Umberto I, Sapienza University of Rome, Rome 00161, Italy; b Breast Unit, Santa Rosa Hospital, Viterbo, Italy; c Breast Center, Department of Surgery, San Giovanni-Addolorata Hospital Rome, Rome, Italy

**Keywords:** Direct-to-implant, Microthane®, Prepectoral position, Capsular contracture, Breast reconstruction, Dash questionnaire

## Abstract

Immediate breast reconstruction (IBR) with polyurethane (PU)-coated implants has usually been performed to mitigate the capsular contracture (CC) rate, particularly in patients requiring post-mastectomy radiotherapy (PMRT). This retrospective study aimed to evaluate the long-term outcomes of direct-to-implant (DTI) reconstruction using Microthane® PU-coated implants placed prepectorally without additional devices.

A total of 143 consecutive patients underwent mastectomy, followed by IBR with Microthane® implants in the prepectoral position. Demographic and clinical data, including complications and CC incidence, were retrospectively collected. Statistical analyses comprised descriptive statistics, Kaplan-Meier survival curves, and the Disabilities of the Arm, Shoulder, and Hand (DASH) questionnaire for patient-reported outcomes.

Over a median follow-up of 4.5 years, 18.9 % of patients experienced at least one complication, with adjuvant radiotherapy being the only factor significantly associated with increased complications (*p* = 0.0229). Kaplan-Meier analysis indicated a complication-free survival rate of 57.85 % for the patient and 58.00 % for the implant, and a contracture-free survival rate of 79.46 % and 81.56 %, respectively. Severe CC (Baker grade III/IV) occurred only in 9.1 % of patients, including several who underwent adjuvant radiotherapy (*p* = 0.0215). These rates remain notably lower than those historically reported with textured implants. Moreover, DASH scores revealed minimal disability (an overall mean score of 8.17 %), reflecting favorable patient-reported outcomes.

Microthane® breast implants in a prepectoral position demonstrated low CC rates and high patient satisfaction, even among those receiving radiotherapy, benefiting from all the prepectoral surgical approaches. In addition, this technique obviates the need for supplementary devices such as meshes or acellular dermal matrices (ADMs), thereby reducing costs, operative times, and potential complications.

The large cohort size and substantial follow-up strengthen these findings. The results support prepectoral reconstruction with Microthane® implants as a safe and effective option in breast reconstruction, offering both aesthetic and functional benefits.

## Introduction

Immediate breast reconstruction (IBR) with implants has been an essential component of post-mastectomy breast restoration since the first augmentation mammoplasty performed by Cronin and Gerow in 1962.^1^ Over the decades, implant-based reconstruction has evolved significantly, yet capsular contracture (CC) remains the most common complication, affecting both functional and aesthetic outcomes.^2^^,^^3^ CC is a fibrotic response to the foreign body, leading to excessive capsule formation around the implant, which in severe cases results in pain, deformity, and the need for surgical revision.^4^ Various implant surfaces have been developed to mitigate CC, including textured and polyurethane (PU)-coated implants. PU-coated implants were introduced in the 1970s as a potential solution to reduce CC incidence by inhibiting the organization of myofibroblasts and minimizing capsule formation. Several studies have suggested that PU implants are associated with a lower CC rate compared to textured implants, particularly in high-risk patients undergoing post-mastectomy radiation therapy (PMRT).^5^^,^^6^ Despite these potential advantages, concerns have been raised about long-term biocompatibility and biodegradation of PU-coated implants.^7^

The impact of radiation therapy on CC development is well documented, with PMRT significantly increasing the risk of severe contracture.^8^ This makes the choice of implant surface a crucial factor in reconstructive planning. While some studies suggest that PU implants maintain their advantage even in irradiated patients,^9^ comparative long-term data remain limited. A previous retrospective study conducted by the authors^10^ analyzed the incidence and severity of CC following IBR with PU versus textured implants, demonstrating a significantly lower CC rate in the PU group. However, further research is needed to confirm these findings and establish the long-term benefits of PU-coated implants in reconstructive surgery.

This study aims to expand upon prior research by retrospectively evaluating CC incidence in patients undergoing IBR exclusively with PU Microthane® breast implants, all in the prepectoral position. By analyzing a consecutive cohort, we seek to provide further insight into the potential advantages of PU implants, particularly in the context of PMRT and specifically in the prepectoral position with Microthane® implants without the use of additional devices (meshes or acellular dermal matrix).

In this study, we aim to demonstrate that Microthane® implants in the prepectoral position provide all the advantages of prepectoral reconstruction while leveraging the benefits of polyurethane coating. This approach eliminates the need for additional devices, such as meshes or ADMs, which may only contribute to increased costs, prolonged operative times, and potentially higher complication rates.^11^

## Materials and methods

### Study design

One hundred and forty-three consecutive patients undergoing mastectomy and one-stage DTI breast reconstruction with Microthane® implants placed in the prepectoral position were retrospectively collected. All the surgeries were performed at the Breast Center, San Giovanni-Addolorata Hospital, Rome, Italy, from January 2018 to June 2024. Our Institution is fully accredited with the Regional Health System and received the European BCCERT Certification in 2017.

The Institutional Review Board approved this study.

Exclusion criteria for this cohort study were delayed or two-stage breast reconstruction, autologous tissue reconstruction, the use of textured implants, smooth implants, saline implants, synthetic meshes or acellular dermal matrix (ADM), previous surgery, or breast implant placed in subpectoral position.

The following characteristics were included: patient age; patient stage; smoking status; presence of diabetes; diagnosis; tumor grade; major and minor surgical complications; neoadjuvant treatments; need for chemotherapy; need for adjuvant radiotherapy, and other characteristics as shown in Table 1. Prior RT refers to radiotherapy following breast-conserving surgery before recurrence, whereas neoadjuvant RT indicates preoperative radiotherapy for tumor downstaging before mastectomy and reconstruction.

**Table 1:** Demographic characteristics of patients, stratified by presence of complications.

	Complication *N* = 27	No complication *N* = 116	Overall *N* = 143	*p*-value
Age (years)	58.5 (0.5)	58.7 (0.5)	58.6 (0.5)	0.1598
BMI (kg/m^2)	27.1 (2.7)	27.1 (2.6)	27.1 (2.6)	0.9086
Smoking status				0.3197
ex	2 (7.4 %)	6 (5.2 %)	8 (5.6 %)	.
no	18 (66.7 %)	92 (79.3 %)	110 (76.9 %)	.
yes	7 (25.9 %)	18 (15.5 %)	25 (17.5 %)	.
Diabetes				0.0704
ex	0	1 (0.9 %)	1 (0.7 %)	.
no	18 (66.7 %)	98 (84.5 %)	116 (81.1 %)	.
yes	9 (33.3 %)	17 (14.7 %)	26 (18.2 %)	.
Stage				1
LABC	7 (25.9 %)	28 (24.1 %)	35 (24.5 %)	.
Metastatic	0	2 (1.7 %)	2 (1.4 %)	.
Missing	0	3 (2.6 %)	3 (2.1 %)	.
early stage breastcancer	18 (66.7 %)	76 (65.5 %)	94 (65.7 %)	.
in situ	2 (7.4 %)	7 (6.0 %)	9 (6.3 %)	.
RT prior surgery				1
no	24 (88.9 %)	102 (87.9 %)	126 (88.1 %)	.
yes	3 (11.1 %)	14 (12.1 %)	17 (11.9 %)	.
Neoadjuvantchemotherapy				1
no	23 (85.2 %)	99 (85.3 %)	122 (85.3 %)	.
yes	4 (14.8 %)	17 (14.7 %)	21 (14.7 %)	.
Adjuvantchemiotherapy				0.723
no	16 (59.3 %)	73 (62.9 %)	89 (62.2 %)	.
yes	11 (40.7 %)	43 (37.1 %)	54 (37.8 %)	.
Neoadjuvantradiotherapy				0.3473
no	27 (100.0 %)	109 (94.0 %)	136 (95.1 %)	.
yes	0	7 (6.0 %)	7 (4.9 %)	.
Adjuvantradiotherapy				0.0229
no	14 (51.9 %)	86 (74.1 %)	100 (69.9 %)	.
yes	13 (48.1 %)	30 (25.9 %)	43 (30.1 %)	.

Note: For continuous variables, values enclosed in brackets represent the standard deviation (SD).

The quality of life of patients and their level of satisfaction following this specific surgical technique with this type of breast implants in a prepectoral position were measured through the DASH questionnaire.

### Surgical technique

All patients underwent mastectomy followed by immediate direct-to-implant (DTI) breast reconstruction with the placement of PU-coated implant in a prepectoral pocket. In all cases, a closed-suction drain was placed and secured externally to the skin. The PU-coated implants used in this study were exclusively Microthane® implants manufactured by Polytech (POLYTECH, Dieburg, Germany).

### Statistical analysis

The analysis was conducted at both the subject and implant levels. Demographic (e.g., age, BMI, smoking habits) and medical data (e.g., prior treatments, comorbidities) were analyzed descriptively, with continuous variables presented as mean and standard deviation (SD) and categorical variables as counts and frequencies. Missing data were not imputed.

To identify significant differences, independent-samples t-tests were used for continuous variables, and Chi-square or Fisher's exact tests (for small expected frequencies) for categorical variables. Follow-up time was calculated from the first surgery to the last follow-up, with “Time to Complication” and “Time to CC” defined as the time to the first complication or CC diagnosis. Implants/patients without complications were censored at the last follow-up. Kaplan-Meier survival curves were generated.

The DASH questionnaire consists of 38 items, divided into three sections: the main module (30 items), the work module (four items), and the sports/recreational activities module (four items). The DASH (Disabilities of the Arm, Shoulder, and Hand) score ranges from 0 % to 100 %, with higher scores indicating greater disability in performing daily activities. The score for each patient was calculated using the following formula:

$$\left[\frac{\sum responses}{number\;of\;completed\;responses}-1\right]x\;25$$

DASH scores were computed separately for each module and for the total questionnaire. The average score for each module and overall was then computed across all patients.

Statistical significance was defined as *p* < 0.05. All statistical analyses and data processing were performed using Statistical Analysis Systems (SAS®) Software (release 9.4).

## Results

This study encompassed a total of 143 patients who underwent immediate, direct-to-implant (DTI) breast reconstruction with polyurethane foam coated implants (Microthane® POLYTECH, Dieburg, Germany) following mastectomy. Microthane® implants were all inserted in the prepectoral position.

The median follow-up was 1630 days (approximately 4.5 years), ranging from 1491 days (4.1 years) to 2043 days (5.6 years). The overall cohort of patients had a mean age of 58.6 years (SD = 0.5) and a mean body mass index (BMI) of 27.1 kg/m² (SD = 2.6). Key baseline characteristics, including smoking status, diabetic status, and cancer stage, were described in Table 1**.** This table demonstrates that the collected demographic characteristics were comparable between the two cohorts of patients: those who developed complications (27 patients) and those who did not (116 patients), as reflected by the numerous non-significant differences in these variables. The unique variable that significantly influenced the onset of complications was the administration of adjuvant radiotherapy (*p*-value = 0.0229).

Out of the 143 patients, 27 (18.9 %) experienced at least one complication, with a total of 37 complication events recorded. Table 2 describes the breakdown of these complications.

**Table 2:** Description of the 37 complications registered.

Complication	Frequency
Contracture III/IV	14
extrusion	4
Implant removal	7
radiodermatitis	3
Revision surgery	3
NAC necrosis	1
Skin necrosis	3
Suspected rupture	1
Infection	1

Note: A patient can experience more than one complication.

**Figure 1:**
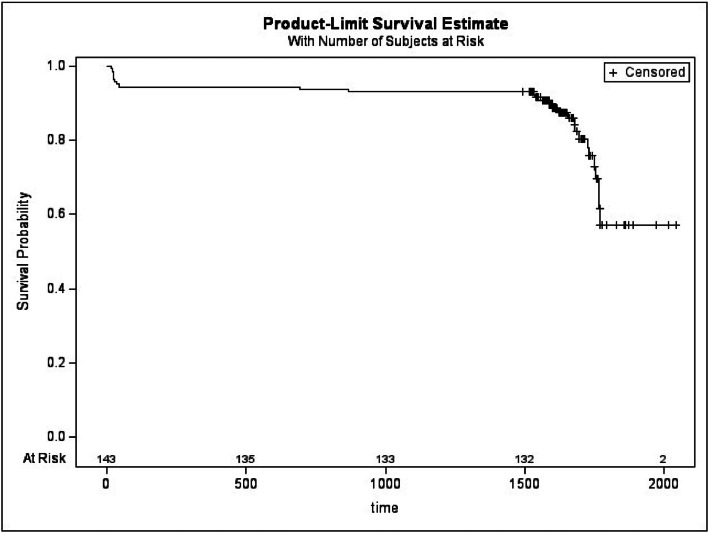
Kaplan-Meier curve of the insurgence of complications by subjects.

**Legend**: + represents censored observations: patients that were complication-free at the end of the follow-up; “at risk” is the number of patients that are still observed at different time points.

**Figure 2:**
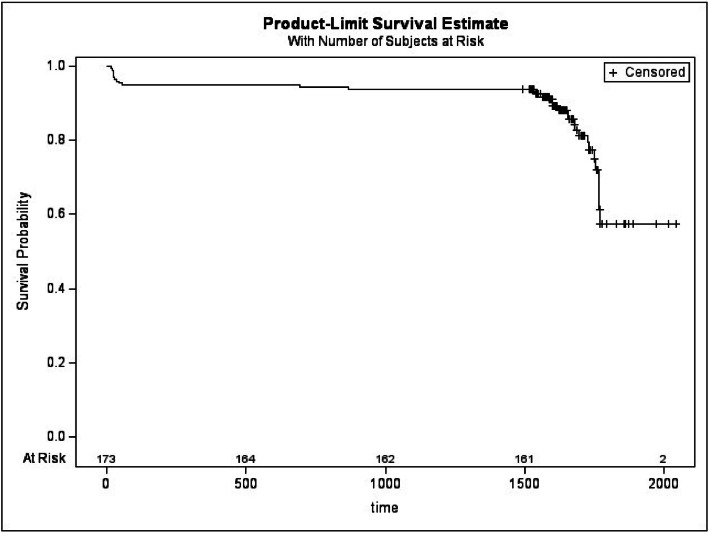
Kaplan-Meier curve of the insurgence of complications by implants.

**Legend**: + represents censored observations: implants that were complication-free at the end of the follow-up.

Kaplan-Meier analysis provided further insight, showing a complication-free survival probability of 57.85 % at the patient level (Figure 1) and 58.00 % at the implant level (Figure 2), at the end of the follow-up period. These survival estimates underscore the importance of long-term surveillance in this kind of patient population.

Focusing specifically on the complication of capsular contracture (CC), 13 patients (9.1 % incidence) experienced severe CC enough to be classified as Baker grade III/IV, with one patient registering CC in both implants (totaling 14 events).

When comparing patients with and without CC, no statistically significant differences were observed in all the variables, except for adjuvant radiotherapy (*p*-value = 0.0215), as described in Table 3.

**Table 3:** Demographic characteristics of patients, stratified by presence of capsular contracture.

	Capsular contracture *N* = 13	No capsular contracture *N* = 130	Overall *N* = 143	*p*-value
Age (years)	58.5 (0.5)	58.7 (0.5)	58.6 (0.5)	0.1717
BMI (kg/m^2)	26.8 (3.2)	27.2 (2.5)	27.1 (2.6)	0.6042
Smoking status				0.8664
ex	1 (7.7 %)	7 (5.4 %)	8 (5.6 %)	.
no	10 (76.9 %)	100 (76.9 %)	110 (76.9 %)	.
yes	2 (15.4 %)	23 (17.7 %)	25 (17.5 %)	.
Diabetes				0.7054
no	10 (76.9 %)	107 (82.3 %)	117 (81.8 %)	.
yes	3 (23.1 %)	23 (17.7 %)	26 (18.2 %)	.
Stage				1
LABC	3 (23.1 %)	32 (24.6 %)	35 (24.5 %)	.
Metastatic	0	2 (1.5 %)	2 (1.4 %)	.
Missing	0	3 (2.3 %)	3 (2.1 %)	.
early stage breastcancer	9 (69.2 %)	85 (65.4 %)	94 (65.7 %)	.
in situ	1 (7.7 %)	8 (6.2 %)	9 (6.3 %)	.
RT prior surgery				0.6542
Missing	0	0	0	.
no	11 (84.6 %)	115 (88.5 %)	126 (88.1 %)	.
yes	2 (15.4 %)	15 (11.5 %)	17 (11.9 %)	.
Neoadjuvantchemotherapy				1
Missing	0	0	0	.
no	11 (84.6 %)	111 (85.4 %)	122 (85.3 %)	.
yes	2 (15.4 %)	19 (14.6 %)	21 (14.7 %)	.
Adjuvantchemiotherapy				0.2389
Missing	0	0	0	.
no	6 (46.2 %)	83 (63.8 %)	89 (62.2 %)	.
yes	7 (53.8 %)	47 (36.2 %)	54 (37.8 %)	.
Neoadjuvantradiotherapy				1
Missing	0	0	0	.
no	13 (100.0 %)	123 (94.6 %)	136 (95.1 %)	.
yes	0	7 (5.4 %)	7 (4.9 %)	.
Adjuvantradiotherapy				0.0215
Missing	0	0	0	.
no	5 (38.5 %)	95 (73.1 %)	100 (69.9 %)	.
Yes	8 (61.5 %)	35 (26.9 %)	43 (30.1 %)	.

**Note**: For continuous variables, values enclosed in brackets represent the standard deviation (SD).

**Figure 3:**
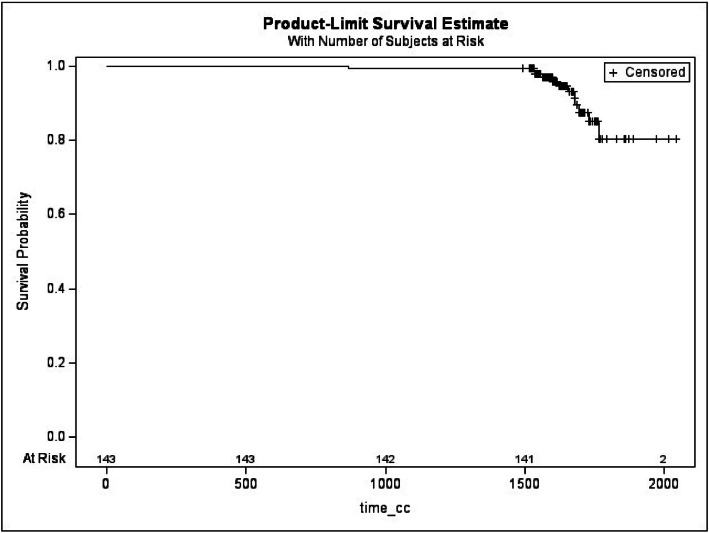
Kaplan-Meier curve of the insurgence of capsular contracture by subjects.

**Legend**: + represents censored observations: patients that were complication-free at the end of the follow-up; “at risk” is the number of patients that are still observed at different time points.

**Figure 4:**
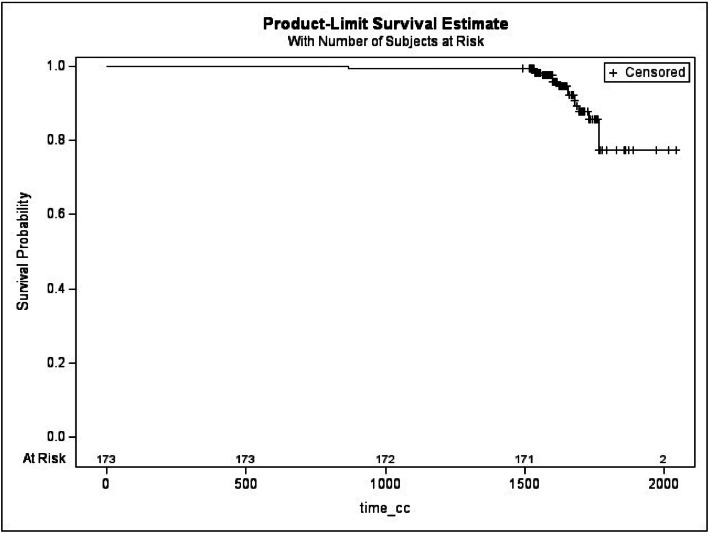
Kaplan-Meier curve of the insurgence of capsular contracture by implants.

**Legend**: + represents censored observations: implants that were complication-free at the end of the follow-up; “at risk” is the number of implants that are still observed at different time points.

Kaplan-Meier curves for CC revealed a contracture-free survival probability of 79.46 % by patient (Figure 3) and 81.56 % by implant (Figure 4) at the end of follow-up. These data suggest that while the overall rate of severe CC with Microthane® implants remained, as described in the literature multiple times, relatively low, its occurrence is a critical factor when considering long-term implant performance, as like in this study.

The statistical analysis further explored the influence of adjuvant radiotherapy on both overall complications and CC. When stratifying patients based on adjuvant radiotherapy as described in Table 4, patients who received adjuvant radiotherapy (*n* = 43) had a significantly higher complication rate (30.2 %) compared to those who did not (14.0 %; *p* = 0.0229). Similarly, the incidence of CC was markedly higher in the adjuvant radiotherapy group (18.6 % versus 5.0 %; *p* = 0.0215).

**Table 4:** Influence of adjuvant radiotherapy on overall complications and capsular contracture.

	Adjuvantradiotherapy *N* = 43	No adjuvant radiotherapy *N* = 100	Overall *N* = 143	*p*-value
Complication				0.0229
Yes	13 (30.2 %)	14 (14.0 %)	27 (18.9 %)	.
No	30 (69.8 %)	86 (86.0 %)	116 (81.1 %)	.
Capsular contracture				0.0215
Yes	8 (18.6 %)	5 (5.0 %)	13 (9.1 %)	.
No	35 (81.4 %)	95 (95.0 %)	131 (90.9 %)	.

When combining neoadjuvant and adjuvant radiotherapy (number of patients = 47), as three patients underwent both neoadjuvant and adjuvant radiotherapy, and comparing these patients to those who did not receive any form of radiotherapy (n° = 96), the overall complication rate did not reach the conventional statistical significance (*p*-value = 0.0605. However, the difference in CC rates remained statistically significant (*p*-value = 0.0299), even if the incidence rates never reached the extremely high value when textured implants are used Table 5, Table 6..

**Table 5:** Influence of adjuvant and neoadjuvant radiotherapy on overall complications and capsular contracture.

	Radiotherapy *N* = 47	No radiotherapy *N* = 96	Overall *N* = 143	*p*-value
Complication				0.0605
Yes	13 (27.7 %)	14 (14.6 %)	27 (18.9 %)	.
No	34 (72.3 %)	82 (85.4 %)	116 (81.1 %)	.
Capsular contracture				0.0299
Yes	8 (17.0 %)	5 (5.2 %)	13 (9.1 %)	.
No	39 (83.0 %)	91 (94.8 %)	130 (90.9 %)	.

**Table 6:** Results from DASH questionnaire.

Module	Percentages
Main module	7.83 %
Work module	8.74 %
Sport/recreational module	10.18 %
Overall score	8.17 %

This study also provided also important information coming from all 143 patients. Patient-reported outcomes were measured using the Disabilities of the Arm, Shoulder, and Hand (DASH) questionnaire, which is comprised of 38 questions across three modules: a main module (30 questions), a work module (four questions), and a sports/recreational module (four questions). The DASH score is expressed as a percentage, with higher scores indicating greater disability. All patients answered all items of the DASH questionnaire, so there were no missing values. In this cohort, the mean scores were:

These low scores indicate minimal disability and a favorable quality of life for most of the patients. Notwithstanding the optimal overall low scores, certain items revealed more significant difficulties in only three patients. One patient reported extreme stiffness of the arm, shoulder, or hand (Item 28), another patient strongly agreed with feeling less capable and confident due to their upper limb issues (Item 30), and the third patient was unable to use their customary technique for playing an instrument or engaging in sports (Item 35). Notably, Item 35 also had the highest number of responses indicating “notable difficulty” (reported by seven patients), suggesting that while most patients function well, a small subset may experience specific challenges in more demanding activities.

## Discussion

The findings of our study reinforce the benefits of prepectoral breast reconstruction with PU-coated implants, particularly in achieving favorable aesthetic outcomes and reducing complication rates. This technique provides significant advantages for patients and surgeons, given the appropriate clinical conditions. Unlike subpectoral reconstruction, the prepectoral approach preserves the integrity of the pectoralis major muscle, thereby avoiding animation deformity, minimizing postoperative pain, and expediting recovery time, all of which contribute to improved patient satisfaction.^12^^,^^13^ The polyurethane coating of PU-coated implants, with its “Velcro effect,” promotes superior adherence to surrounding tissues, mitigating the risks of implant displacement and seroma formation. Unlike textured implants, which necessitate additional supportive devices such as ADMs or synthetic meshes, Microthane® implants function effectively without these adjuncts. By eliminating the need for costly and time-consuming additional devices, this technique optimizes surgical efficiency, reduces the financial burden, and minimizes the potential for device-related complications such as infection or delayed wound healing.^14^ Moreover, the use of Microthane® implants has emerged as a less expensive and more convenient alternative in certain European countries, further supporting their adoption in clinical practice.^15^

One of the most significant findings of this study is the confirmation of a low incidence of CC,^16^, ^17^ even among oncologic patients who underwent post-mastectomy radiotherapy (PMRT).^18^Capsular contractureis one of the most concerning complications in implant-based breast reconstruction, as it can severely affect aesthetic and functional outcomes. Our results demonstrate that Microthane® implants substantially decrease the risk of severe CC (Baker grade III/IV), likely due to the unique biomechanical properties of the polyurethane coating. This is particularly relevant because, as previous studies have shown,PMRT is widely recognized as a major risk factor for CC development. The lower CC rates observed in our study, even among irradiated patients, suggest that the use of PU-coated implants represents an effective strategy for minimizing this complication compared to traditional textured implants.

Our study demonstrates that combining Microthane® implants with the prepectoral surgical approach provides aesthetic benefits by reducing CC and minimizing the risk of animation deformity. These aesthetic advantages enhance patient satisfaction and quality of life, particularly in the context of implant-based breast reconstruction, including cases involving radiotherapy.

Moreover, according to recent retrospective studies, PU implants are associated with fewer short-term complications and lower reconstructive failure rates than ADM-covered implants, reinforcing their clinical benefits.^15^ Such understanding is pivotal in improving surgical outcomes. Careful patient selection is essential when proposing prepectoral DTI reconstruction. Factors such as obesity, large and ptotic breasts, and increasing mastectomy weight are commonly described risk factors for complications in implant-based reconstruction. However, recent studies suggest that prepectoral implant placement may be a better reconstructive option than the subpectoral plane in high BMI populations. This observation underscores the importance of individualized patient assessment in optimizing surgical outcomes and minimizing risks.

Despite the strengths of our study, certain limitations should be acknowledged. First, its retrospective nature restricts the ability to control for confounding variables. Furthermore, the absence of a control group prevents direct comparisons with alternative reconstructive techniques, such as subpectoral implant placement or the use of different implant types. However, including a large patient cohort with a substantial follow-up period enhances the robustness of our findings. In the future, randomized studies will provide higher-level evidence to further validate this approach's benefits. A distinctive feature of our study is the integration of patient-reported outcomes, as assessed using the DASH questionnaire. The DASH scores in our cohort indicated minimal disability and a favorable quality of life, underscoring the functional advantages of prepectoral reconstruction. The preservation of the pectoralis major muscle is a key factor in maintaining upper limb function, a benefit that is particularly important for daily activities and overall well-being. Moreover, there is a growing appreciation for the high levels of patient satisfaction reported in our study, highlighting the aesthetic advantages of this technique, particularly the natural breast contour and the absence of animation deformity.

## Conclusion

Our study undoubtedly confirms that prepectoral breast reconstruction with Microthane® implants is a reliable procedure, demonstrating a low complication rate and a significantly reduced incidence of CC. The unique characteristics of the PU foam allow for correct implant positioning without the need for additional mechanical support, which, in turn, further contributes to the efficiency and safety of the procedure. Given these advantages, PU-coated implants represent a valuable option in implant-based breast reconstruction, particularly in patients at higher risk of complications. These advantages enhance patient satisfaction and quality of life, particularly in the context of implant-based breast reconstruction, including cases involving radiotherapy. While further high-quality prospective studies are encouraged to evaluate differences among various prepectoral reconstruction techniques, our findings spotlight the clinical benefits of this approach. Notably, despite the need for additional comparative studies, patient-reported outcomes in our study revealed excellent satisfaction levels, reinforcing the aesthetic and functional advantages of PU-coated implants in prepectoral reconstruction. Therefore, since its introduction, this approach has continued to show promising results in improving patient outcomes.

## Ethical approval

The Institutional Review Board approved this study.

## Funding

None

## Declaration of competing interest

None declared.
